# Dissecting Source-Sink Relationship of Subtending Leaf for Yield and Fiber Quality Attributes in Upland Cotton (*Gossypium hirsutum* L.)

**DOI:** 10.3390/plants10061147

**Published:** 2021-06-04

**Authors:** Naimatullah Mangi, Mian Faisal Nazir, Xiaoyan Wang, Muhammad Shahid Iqbal, Zareen Sarfraz, Ghulam Hussain Jatoi, Tahir Mahmood, Qifeng Ma, Fan Shuli

**Affiliations:** 1 State Key Laboratory of Cotton Biology, Institute of Cotton Research, Chinese Academy of Agricultural Sciences (ICR, CAAS), Anyang 455000, China; manginaimatullah2014@gmail.com (N.M.); mfn121@hotmail.com (M.F.N.); shahidkooria@gmail.com (M.S.I.); zskpbg@gmail.com (Z.S.); tahirmtaha@hotmail.com (T.M.); 2 Anyang Institute of Technology, College of Biology and Food Engineering, Anyang 455000, China; 20160539@ayit.edu.cn; 3 Cotton Research Station, Ayub Agricultural Research Institute, Faisalabad 38850, Pakistan; 4 Department of Agriculture, Mir Chakar Khan Rind University, Sibi 82000, Pakistan; ghulam.hussain@mckru.edu.pk

**Keywords:** source-sink relationship, subtending leaf, cotton boll, multivariate analysis, upland cotton

## Abstract

Photosynthesis as a source is a significant contributor to the reproductive sink affecting cotton yield and fiber quality. Moreover, carbon assimilation from subtending leaves adds up a significant proportion to the reproductive sink. Therefore, this study aimed to address the source-sink relationship of boll subtending leaf with fiber quality and yield related traits in upland cotton. A core collection of 355 upland cotton accessions was subjected to subtending leaf removal treatment effects across 2 years. The analysis of variance suggested a significant effect range in the source-sink relationship under subtending leaf removal effects at different growth stages. Further insight into the variation was provided by the correlation analysis and principal component analysis. A significant positive correlation between different traits was observed and the multivariate analysis including hierarchical clustering and principal component analysis (PCA) categorised germplasm accessions into three groups on the basis of four subtending leaf removal treatment effects across 2 years. A set of genotypes with the lowest and highest treatment effects has been identified. Selected accessions and the outcome of the current study may provide a basis for a further study to explore the molecular mechanism of source-sink relationship of boll subtending leaf and utilization of breeding programs focused on cotton improvement.

## 1. Introduction

Cotton, *Gossypium* spp., is widely accepted as the major fiber source globally and is cultivated across most of the tropical and subtropical parts of the world [1]. Among the prominent cotton producers, China, USA, India, Pakistan, and Uzbekistan are considered major shareholders [2,3]. The major limitations in cotton productivity are the biotic and abiotic stresses, including drought, heat, salinity, insect pests, diseases, and weeds, particularly during the early development stages [4]. Cotton is generally considered a resilient crop against stressors. However, viral infestations such as the Cotton Leaf Curl Virus Disease (CLCuD) have become major threats during the last two decades due to heavy infestations across South Asia and some parts of the USA [5]. Additionally, cotton growth and development may be highly challenged due to future climate change scenarios [6]. Upcoming climate changes are likely to play a prominent role in the change in cotton crop productivity patterns either positively or/and negatively. Fluctuating temperatures may significantly influence cotton growth and development due to a higher impact on respiration, transpiration rate, and photosynthesis, ultimately influencing fruit production [7].

The fiber quality attributes, including fiber length, strength, fineness, uniformity, and elongation, are the key desirable factors for optimizing textile processing and producing high-quality textile products [8]. A higher lint production is also considered as one of the most critical factors among major cotton production systems focusing on enhancing profitability [9]. Several cotton yield components play a significant role in enhancing lint yield, including bolls per plant, boll weight, seeds per boll, and ginning out percentage [10,11]. During the recent era, most of the improved cotton varieties being cultivated face a narrow genetic base due to the extensive unidirectional artificial selection by breeders for yield enhancement [12]. It became a serious concern nowadays, as narrow or limited genetic diversity may lead to a deficient allelic availability for a continued genetic improvement of cotton [13,14]. Hence, it is worth understanding the genetic basis of various domesticated traits and maintaining diversity among species to be utilized as a source in future breeding programs aimed at cotton improvement [14,15,16].

Since fibers in cotton are exclusively acclaimed as natural fibers and are made up of cellulose, with a polysaccharide 1,4-d-glucopyranose structural unit [17]. It is assumed that there must be a fundamental linkage among photosynthate assimilation, storage, and plant growth in terms of their strong relation as a source-sink [18]. Hence, a potential connection between carbon utilization and transport from photosynthate assimilation source tissues to the non-photosynthetic tissues is considered a sink [19]. Furthermore, fiber quality in cotton, including length, strength, and fineness, is extensively influenced by the photosynthate concentration in assimilates. During favorable conditions in the presence of adequate nutrients, light, and moisture, the cotton boll under-development may be assumed to acquire almost 60% or more assimilates required for fiber growth solely through subtending the leaf nearest to it [20,21]. Translocation becomes essential through other plant parts to supplement the remaining 40% development [22]. Therefore, an early vegetative growth is preferred in order to overcome the increased competition of different bolls as sinks are required to get translocated during the upcoming reproductive phase. Many published reports concluded that until the 12th node, the vegetative growth rate is usually enhanced, resulting in the derived carbohydrate transport towards reproductive units (bolls), unless any significant translocation among branches is accomplished [23,24].

Around 60–87% carbon in mature cotton boll is detained through CO_2_ assimilation across the boll development, and as this process is carried out, the subtending leaf has a significant contribution for photosynthate accumulation, ultimately leading to increased biomass in seed cotton [20,21,24]. In this process, bolls and subtending leaves are linked in a sink-and-source-relationship to utilize photosynthate accumulation [25,26]. We can interpret their relationship as coordination among vegetative and reproductive growth phases in cotton with a substantial impact on the yield and quality of cotton produce [27]. Numerous studies on source-sink relationships have reported that an earlier and efficient sink development may lead to a robust and more substantial reproductive growth potential [28]. Higher yields are attributed to the substantial photosynthesis rate within the functional leaves, which is considered a source activity and is essential and effective for efficient photosynthates distribution through the reproductive parts [29,30].

A competition prevails for photosynthates across different plant development stages (Stewart 1989). The leaf is assumed to play an essential role in plant growth and development and is known as “powerhouse” in the plants due to its functionality as food synthesis in the presence of chlorophyll (Gitelson et al. 2003). Numerous leaf traits have been reported to show a substantially significant role in yield [31]. Considering the cotton plant as a reference, the leaf life cycle starts with bud initiation, acting as a carbohydrate exporter within a few days [32]. As the boll emerges, it starts extracting its maximum nutrition mostly from its subtending leaf [33].

The multivariate analysis has been extensively used as an important and mostly primarily acceptable method to estimate genetic variability and diversity in a more accurate manner [34]. It can also determine possible patterns of dissimilarities and potential genetic relationships within a specific germplasm collection [35]. The multivariate analyses have been extensively utilized globally by different crop improvement programs [36] on various crops such as wheat [35], maize [37,38], and sorghum [39].

The present-day improved cotton cultivars are assumed to be developed using limited germplasm sources as parents [40]. Due to the lack of extensive studies to explore the relationship between source-sink so far, this has made it difficult for a clear understanding in this regard and we are still handicapped in developing an optimal rationale for source-sink relationship manipulations. Hence, the current study has been planned to investigate the source-sink relationship of subtending leaves with boll influencing fiber quality and yield traits of upland cotton accessions during the years 2018 and 2019. The main objectives of the current study were aimed at: (a) The assessment of variability and diversity of the studied germplasm in response to the source-to-sink relationship; (b) Exploration of genotypes expressing minimum and maximum source-sink dependency. This study will be helpful in providing a deep insight into the source-sink relationship of subtending leaf with its boll and effect on yield and fiber quality. It will also provide a baseline for possible source-to-sink manipulations for cotton improvement yield and fiber quality.

## 2. Materials and Methods

A diverse collection containing 355 upland cotton accessions taken from the gene bank of the Institute of Cotton Research of CAAS has been considered plant material and utilized for the study. The collection comprised 331 cultivars developed and originated from China, whereas 24 cultivars were introduced from different countries. The Chinese accessions have been further subdivided into the five groups concerning the ecological areas of their origin, including 162 accessions from the Yellow River region (YR), 51 accessions from the Yangtze River Region (YZR), 98 accessions from the northwest in the land region (NW), 20 accessions from the Liaoning province (LN), and the foreign group consisted of 20 accessions from the USA, while four accessions were from countries of Central Asia. These cotton accessions have been planted following a triplicated randomized complete blocked design (RCBD) in the factorial arrangement at the field area of CRI, Anyang Henan during the sowing season in 2018 and 2019. Sowing was carried out on 30 April during both years viz., 2018 and 2019. The plot size was maintained as 3.5 m length of each accession in each replication and for each treatment.

After germination, thinning was carried out to maintain the plant population by keeping 10 plants in each row. The chemical control was applied at peak flowering and the boll setting period, whereas all other agronomic and plant protection practices were practiced following local recommendations. Five guarded plants from each line were selected randomly and tagged to be considered for further study. The start of the blooming flower tagging in all the experimental plots was carried out beginning from July 10, onward to August 10. Each flower during this period has been tagged from its blooming date. The boll subtending leaf removal has been carried out for the tagged leaves, as they reach 35 the days’ bolls represented as T_1_, 40 days’ bolls as T_2_, 50 days’ bolls as T_3_, 60 days’ bolls as T_4_, and one check as the control where the subtending leaf was kept unremoved (Table 1). The subtending leaf removal was carried out by hand on 20 bolls from each replication per accession from each treatment from the five selected and tagged plants. On maturity, picking was carried out to pick 20 bolls from every five plants for each treatment. The fiber quality for the nine traits was recorded for the picked bolls using a high volume instrument (HVI) in the Laboratory of Quality and Safety Risk Assessment for Cotton Products (Anyang), Ministry of Agriculture, People’s Republic of China. The traits considered for the current study included boll weight (BW), seed weight (SW), ginning outturn (GOT%), fiber weight (FW), fiber length (FL), fiber uniformity (FU), fiber fineness (MIC), fiber strength (FS), and fiber elongations (FE).

The effect of source (subtending leaves) to sink (boll) was estimated by comparing different treatments (removal of subtending leaves at multiple growth stages as shown in Table 1) with the control. Additionally, differences were considered as the source-sink effect of the corresponding trait.

Subtending Leaf *Effect(E) = Control− Treatment* (Mean)

where *E* is the subtending leaf effect or tolerance indice, control is the treatment with unremoved subtending leaf, whereas treatment is used for the corresponding treatment with the removed subtending leaf on different dates post anthesis (DPA).

The effects E1, E2, E3, and E4 were calculated using the same formula as above with the recorded values for each trait under study and their corresponding control values across both years.

### Statistical Analysis

The calculated source-sink effects of 355 accessions for the E1–E4 effects across 2 years, as means of the ramets obtained for each genotype, were subjected to a combined analysis of variance of genotypes and source-sink effects across years using the general linear model (GLM), since the missing data from different replications over the treatments and years did not permit the repeated measures ANOVA for each treatment and year of the experiment. Factors were analyzed individually without interactions to avoid model complications that make it inappropriate. The expected mean squares were based on a random effects model for genotypes, treatments, and years.

The multivariate analysis was performed including basic statistics, PCA, and cluster analysis categorizing genotypes, years, and treatments for the source-sink effect and its relationship to the various variables under study. All the analyses were carried out using two statistical software packages: SAS-JMP Pro 15 (SAS Institute Inc., Cary, NC, USA, 1989–2019) and R 3.4.0.

## 3. Results

**Figure 1:**
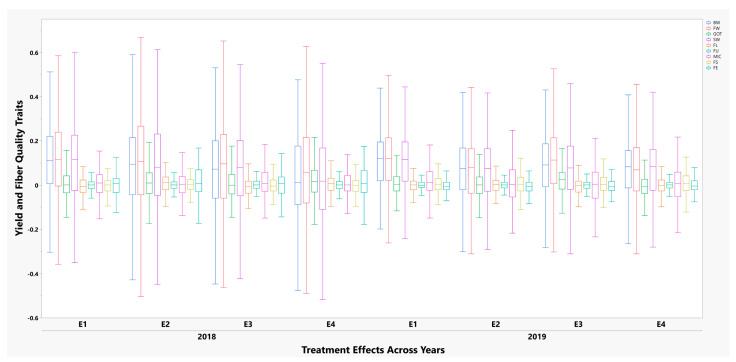
Distribution of four treatment effects of subtending leaf removal on fiber quality and yield related traits among 355 upland cotton accessions across years 2018 and 2019. Legends on the top right in different colors depict nine evaluated phenotypic traits.

The variation existing for the removal of subtending leaf effects in the core germplasm of upland cotton (355 accessions) was estimated using the mixed linear model. The four treatment effects of subtending leaf removal derived for each trait were compared to dissect the source-sink relationship of subtending leaf with the yield and fiber quality. The differences were then termed as a source-sink effect for each trait under study. The summary statistics of the data collected is revealed in Figure 1. It showed that a high range of values existed regarding the removal of subtending leaf treatment effects in year 2018 as compared to 2019. Furthermore, the yield related traits particularly SW, BW, FW, and GOT showed more variation effects across both years in comparison to the fiber quality traits.

The overdispersion parameter estimated by the maximum likelihood using the generalized linear model (GLM) for fiber quality and yield related traits in 355 upland cotton accessions, under four treatment effects (E1–E4) across 2 years (2018 and 2019), are represented as Supplementary Tables S1–S9. From these results, the effect summary and effect tests have been concluded, since ANOVA (Table 2) suggested significant L-R chi-square values along with Log Worth values as represented in the summary effect plots in Table 2 among the genotypes for all traits. Whereas, the treatment effects have been significant for yield traits viz., boll weight (BW), fiber weight (FW), ginning outturn (GOT), and seed weight (SW). In contrast, fiber quality traits viz., fiber length (FL), fiber uniformity (FU), micronaire (MIC), fiber strength (FS), and fiber elongation (FE) showed non-significant treatment effects for the source-sink relationship. Furthermore, ANOVA also depicted a significant variation within the growing seasons viz., 2018 and 2019 for BW, FW, SW, FS, and FE. The significant variation of traits in different growing seasons depicted a significant environmental influence. However, GOT, FL, FU, and MIC did not show significant differences in the two seasons as the source-sink effect.

### 3.1. Correlations

**Figure 2:**
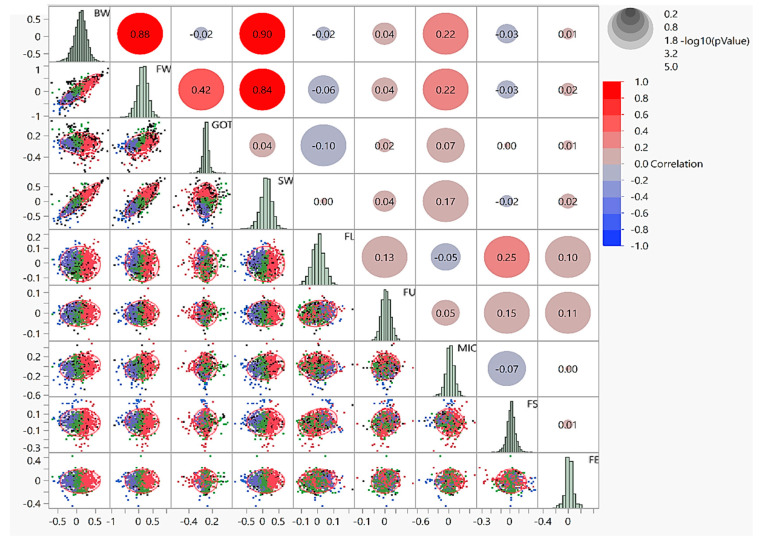
Scatterplot matrix to visualize several attributes by pairwise dependencies of nine fiber quality and yield related traits. The upper triangle matrix represents the correlations among source-sink effects of nine studied traits. Histograms at diagonal depict the shape of frequency distribution for the data of investigated traits, whereas the lower triangle matrix reveals the bivariate density distribution with ellipses between each pair of traits. The legends at the top right corner of the color gradient (red to blue) and the size of circles show the amount of correlation and *log p*-value for significance threshold, respectively.

The correlation and its distribution related to the source-sink effect of the nine traits in question were estimated to reveal their corresponding relationships (Figure 2). The upper triangle of the correlogram depicted correlations among the traits of the source-sink effect, while the lower triangle exhibited a scatterplot matrix of their density distributions. Source-sink effects of investigated traits displayed highly significant (≤0.0001) and high positive correlations between BW and FW (0.88), BW and SW (0.90), as well as FW and SW (0.84). MIC exhibited a significant negative correlation with FL and FS. GOT displayed a highly significant negative correlation with FL. Furthermore, significant negative correlations were observed between various source-sink effects of fiber quality and yield related traits. At the diagonal of the matrix, all the traits represent a normal state of frequency distribution in the shape of an histogram, which fits the data as suitable for the generalized linear model (GLM) with a normal distribution option. The lower triangle is for the depiction of the pairwise density distribution of traits. The narrowness of the ellipse shows the degree of correlation between a particular pair of traits in a specific cell. The narrow shaped ellipse with a diagonally oriented distribution shows the correlation between the paired traits, and the fairly round shaped ellipse with no diagonally oriented distribution shows no correlation.

### 3.2. Principal Component Analysis (PCA)

The principal component analysis (PCA) can be considered as the most acceptable method, utilizing a multivariate approach, carrying out a dimension reduction across independent and interdependent variables retaining only those characteristics from a dataset contributing to its variance [41]. PCA was performed based on the correlation among source-sink effects of yield components and fiber quality traits. Nine PCs have been extracted from nine studied traits (Table S10) through PCA. A total of four PCs have been detected with Eigen values above 1, whereas the remaining components depicted minimal Eigen values.

The first component PC1 contributes to various traits towards variability, concluding that the source-sink effect of FW has a major contribution towards positive loading vectors (0.96435), followed by the source-sink effect of BW (0.94493), FW (0.95579), SW (0.91334), and MIC (0.31909) within the first component. It can be concluded that four major variables mentioned have a strong correlation with the first PC. The source-sink effect of FW has been found to have a strong correlation to this principal component. Hence, we can assume that PC1 can ultimately be considered as a measure to the source-sink effect for yield related traits. However, the remaining traits can be seen with a minimal contribution with positive loadings. PC2 exhibited source-sink effects of FL (0.71787) followed by FS (0.65328), FU (0.54193), FS (65328), and FE (0.33166). PC3 was related to GOT (0.87543) followed by FU (0.32163). PC4 revealed a maximum contribution towards FE (0.70599) and MIC (0.37958) as positive loadings, whereas FS (−0.46201) as negative loadings, respectively (Table S11).

**Figure 3:**
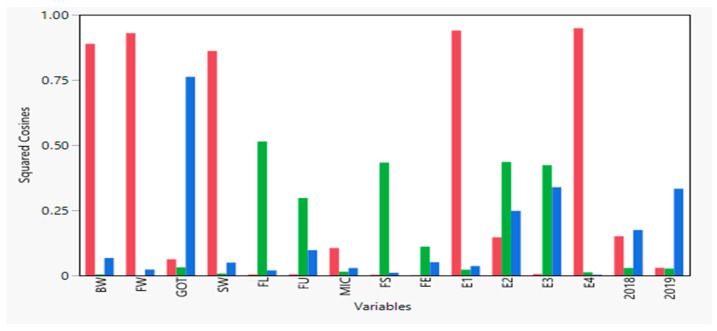
Squared cosines associated with the principal components for the studied traits, treatment effects (E1–E4), and years 2018 and 2019.

As shown by the principal component analysis results, a total of 59.787% variance has been explained by the first three components (PCs). The yield, as well as fiber quality traits, are represented through factor map squared cosines or squared coordinates. It is determined that the high values of squared cosines give a satisfactory contribution to the particular variable. Figure 3 shows that all the yield related traits cover PC1 except for GOT. However, all the fiber quality traits fall in PC2 except MIC, which is in PC1. With regards to the treatment effects, E1 and E4 fell in PC1 while, E2 and E3 fell in PC2. Furthermore, both years got maximum values in PC3.

**Figure 4:**
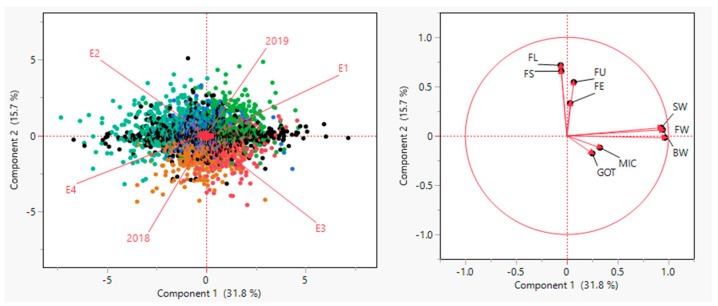
Summary plots with (**left**) biplot between PC1 and PC2 displaying the distribution of 355 upland accessions across treatment effects and years; (**right**) contribution of different traits in variation for genotypes, treatment effects, and years.

In this study, most of the variables got a place nearest to the correlation eclipse in the summary plot, as shown in Figure 4. A significant representation of different variables across the first PC is depicted by the length of originating vectors. Almost all the fiber quality traits viz., FL, FS, FU, and FE except MIC, lied in the direction of PC2, whereas GOT, BW, FW, SW, and MIC lied in the direction of PC1. MIC and GOT are very close to the center with minimum contributions.

**Figure 5:**
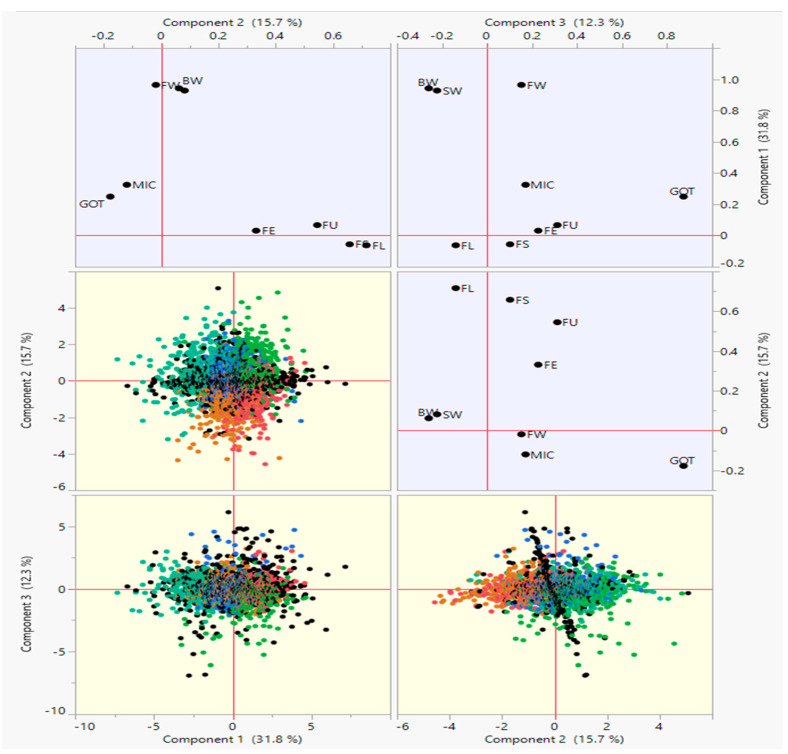
Scatterplot of PC1, PC2, and PC3 displaying the contribution of different traits.

A more elaborative depiction of the traits in three PCs has been presented in Figure 5. In the biplot of PC1 and PC3, fiber yield traits viz., BW, FW, and SW lied close to each other concluding the presence of a high correlation among them. Meanwhile, in PC2 and PC3 biplot, fiber quality traits viz., FS, FE, and FU exhibited more correlation among themselves. These variables can be considered as critical in order to explain the variability in the studied dataset. The fiber fineness as MIC has not shown any relationship with any PC (Figure 5).

The principal component biplot drawn for the studied material indicated considerable variability among the studied treatments (Figure 4). A plot between PC1 and PC2 related to yield and fiber quality traits in a collection of 355 accessions has been clustered into three distinct groups encountering four subtending leaf removal treatment effects (E1–E4). For further clarification and details, the sub-populations have been represented by three different colors based on the yield contribution and fiber quality traits under study. It has been observed that the accessions which have been placed together are considered to have high similarity with each other.

### 3.3. Cluster Analysis

**Figure 6:**
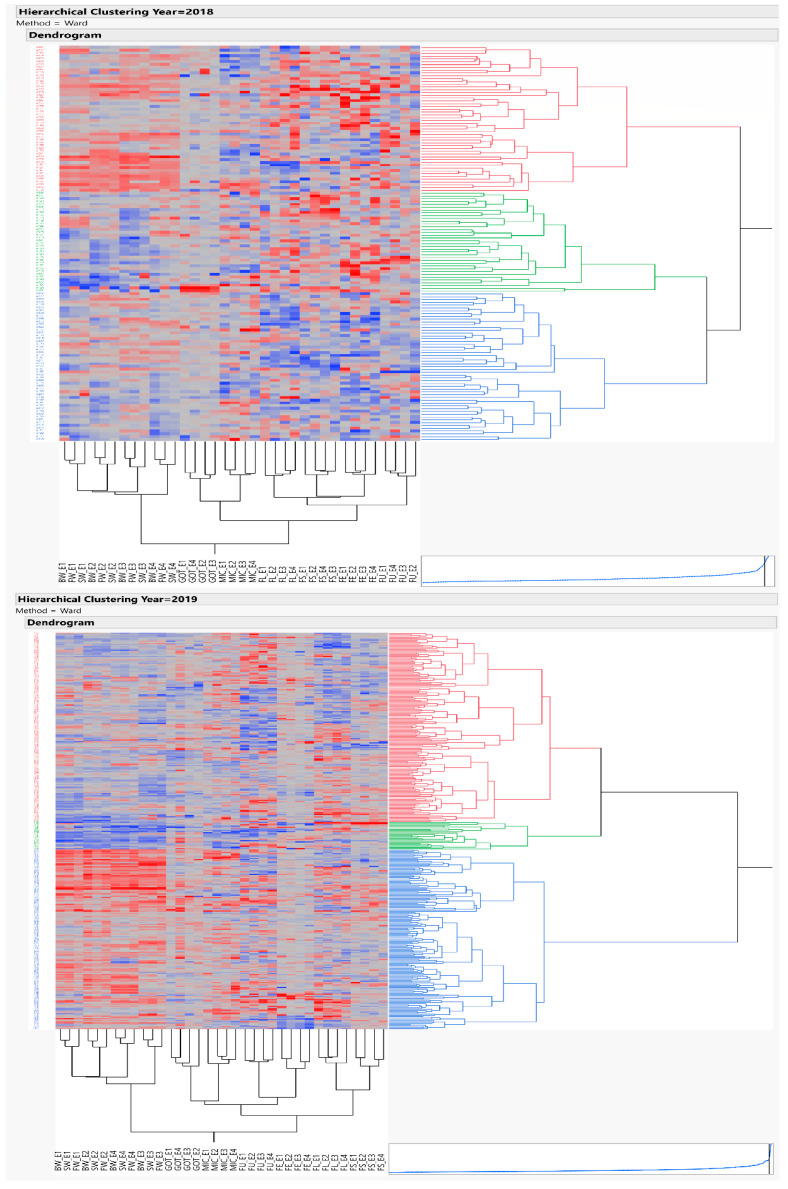
Hierarchical clustering of 355 upland cotton accessions for yield related and fiber quality traits based on treatment effects across years 2018 and 2019.

A scatter plot matrix, represented in Figure 6, has been drawn using factor scores as PC1 and PC2, and depicted a clear pattern for grouping of genotypes by observing three major distinct groups through clustering. The factors’ correspondence was further subjected to agglomerative hierarchical clustering to work out the Euclidean distance matrix through Ward’s method, and a dendrogram has been constructed based on the result. Numerous methods are available for the estimation of diversity within germplasm subjected to various contrasting environments. The two-way clustering has been executed through the AHMC method, resulting in a two-way cluster diagram and constellation plots. A scatterplot matrix has also been drawn to determine the densities composition of the cotton germplasm under different subtending leaf removal treatments at different boll ages. Depending on the clustering, we identified 10 genotypes, five with the high effect of source (subtending leaf) removal and five with the least effect of source (subtending leaf) removal. The selected genotypes have been presented in Table 3. These genotypes can be further utilized in breeding programs to manipulate the source-sink relationship of subtending leaf in upland cotton.

## 4. Discussion

The source-sink relationship in plants is critical for the end-product as yield, and is a complicated phenomenon affecting the cotton yield and fiber quality. To understand the dynamics of the source-sink relationship, the core germplasm of upland cotton accessions was evaluated. The subtending leaf was considered a primary source, affecting the yield and fiber quality in cotton [26]. The effects of subtending leaf removal were subjected to multivariate data across 2 years and four subtending removal treatment effects related to fiber quality and yield traits. In addition, the corresponding variation in core germplasm was evaluated using the generalized linear model (GLM), correlations, PCA, and clustering. It is generally assumed that the subtending leaf is the major contributor to biomass accumulation in cotton bolls [42]. Hence, subtending leaves act as a source for their corresponding bolls, acting as a sink. This source-sink relationship is reflected as the coordination of vegetative and reproductive growth, and cotton development influences yield and fiber quality in cotton [43]. Considering a very basic and simple example, we look at a plant with two organs viz., leaf and root. Leaves are involved in carrying out the photosynthesis process and can be considered the sole source of carbon, whereas roots are sinks for carbon and dependent on the leaf to support their growth and development. In contrast, we can consider roots as sources of nutrients and nitrogen, which they get from the soil and provide to the leaves, acting as net sinks in this scenario. Each plant cell utilizes carbon for respiration, growth of essential metabolites, building up proteins, various enzymes, and other genetic materials [44]. Hence, we can assume that mature leaves involved actively in photosynthesis have a relatively small carbon-sink activity. On the contrary, roots have a small nitrogen-sink activity and higher nitrogen-source activity, making them net sources for nitrogen (Burnett 2019).

Moreover, checking the photosynthesis through subtending leaves has been created by shading across flowering and the boll formation stage [45,46,47]. However, no clear and state-of-the-art experiment was reported to determine the source-sink relationship between subtending leaves with cotton boll, which is ultimately crucial to fiber yield enhancement in cotton [48]. Previously, a few studies have reported that the variation of starch and sucrose in cotton fibers have a remarkable association with fiber quality [49]. The portioning of photosynthates during the boll development stage (ovule or the fiber development) were primarily from the subtending leaves of the boll (Ryser 1992), and the content of sucrose and starch in cotton subtending leaves was higher than hexose [49,50]. Therefore, it is pertinent to examine the content and variation of sucrose and starch that might affect the fiber quality. In our study, all the genotypes depicted considerable variations when analyzed for fiber quality traits of FE, FU, FS, MIC, and FL and yield related traits such as BW, FW, SW, and GOT. The correlation matrix is used to investigate the dependence between multiple variables [51]. The significant positive associations among fiber quality parameters, i.e., FU, FL, FS, FE, and MIC, were observed in our study, which is in line with previously published statistics [52,53]. Similar to earlier findings, the current investigation found negative correlations among yield related and fiber quality traits. For instance, highly significant negative correlations have been exhibited by FE with SW and FS with FW and GOT [54]. However, GOT% revealed a highly significant positive correlation with MIC, as reported previously [55,56]. MIC depicting a significant negative correlation with FL has also been reported previously [57,58]. One of the liable mechanisms behind such negative correlations is repulsive linkage [58]. In these instances, superior genotypes harboring desirable traits related to yield and fiber quality can be utilized as recurrent parents to engulf negative correlations in selection-breeding programs [54]. In previous findings, FS and MIC revealed a positive correlation with each other as in the current study [59]. A biplot analysis has been implemented to understand the multivariate relationships among fiber quality and yield contributing traits across 355 upland cotton accessions concerning the association between germplasm accessions and the observed traits.

## 5. Conclusions

In the current study, insight into the variation of source-sink effects suggested a significant range of variation, as well as a positive correlation between different traits in core germplasm accessions under treatment effects across years. The treatment effect through the principal component analysis and hierarchical clustering methods divided accessions into three groups, based on four treatments across 2-year data. Our results depicted a significant variation among the core germplasm for the treatment effect through yield traits as well as for fiber length, and provide a basis for further studies to explore the source-sink relationship of subtending leaf with yield and fiber quality. A set of cotton accessions with higher treatment effect, which is considered to have a strong source-sink relationship and lower compensation effect along with a set of genotypes exhibiting a lower treatment and higher compensation effect, has been selected from this study. The reported results and selected genotypes based on their source-sink relationships can be further utilized in breeding programs to understand and evaluate the molecular mechanism behind the source-sink relationship in terms of carbon accumulation.

## Figures and Tables

**Table 1:** Description of subtending leaf removal of 355 cotton accessions to determine the source-sink relationship.

Treatment	Description	Number of Entries
Control	No Subtending Leaf Removal	355
T1	Subtending Leaf removal after 35 Days boll age	355
T2	Subtending Leaf removal after 40 Days Boll age	355
T3	Subtending Leaf removal after 50 Days boll age	355
T4	Subtending Leaf removal after 60 Days Boll age	355

**Table 2:** Analysis of variance (ANOVA) using the generalized linear model for the source-sink effect (E1–E4) across 2 years 2018 and 2019.

Trait	Source	DF	L-R Chi Square	Log Worth	Summary Graph	*p*-Value
**BW**	Genotype	354	966.18469	57.516	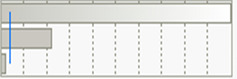	<0.0001 *
	Treatment Effect	3	54.951574	11.153		<0.0001 *
	Year	1	3.1565769	1.121		0.0756
**FW**	Genotype	354	937.32891	53.560	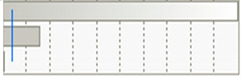	<0.0001 *
	Treatment Effect	3	39.597259	7.887		<0.0001 *
	Year	1	0.7938599	0.428		0.3729
**GOT**	Genotype	354	794.35135	35.114	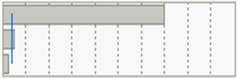	<0.0001 *
	Treatment Effect	3	14.489381	2.637		0.0023 *
	Year	1	4.1162681	1.372		0.0425 *
**SW**	Genotype	354	960.37802	56.714	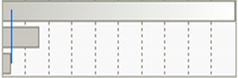	<0.0001 *
	Treatment Effect	3	39.621111	7.892		<0.0001 *
	Year	1	6.0551756	1.858		0.0139 *
**FL**	Genotype	354	1030.1228	66.517	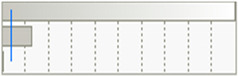	<0.0001 *
	Treatment Effect	3	32.998526	6.492		<0.0001 *
	Year	1	0.6609049	0.381		0.4162
**FU**	Genotype	354	997.69022	61.912	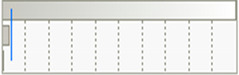	<0.0001 *
	Treatment Effect	3	2.4583405	1.603		0.4829
	Year	1	5.0287601	0.316		0.0249 *
**MIC**	Genotype	354	1081.4485	73.956	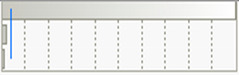	<0.0001 *
	Treatment Effect	3	6.4663942	1.041		0.0910
	Year	1	1.3158987	0.600		0.2513
**FS**	Genotype	354	1073.4359	72.783	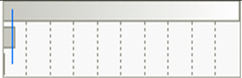	<0.0001 *
	Treatment Effect	3	2.8515984	2.548		0.4151
	Year	1	8.9126018	0.382		0.0028 *
**FE**	Genotype	354	964.29565	57.254	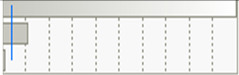	<0.0001 *
	Treatment Effect	3	3.6658411	5.525		0.2999
	Year	1	21.825712	0.523		<0.0001 *

BW: Boll weight; FW: Fiber weight; GOT: Ginning outturn; SW: Seed weight; FL: Fiber length; FU: Fiber uniformity; MIC: Micronair; FS: Fiber strength; and FE: Fiber elongation; DF: Degree-of-freedom; L-R Chi Square: Likelihood ratio for Chi Square. * Significant at *p* < 0.01.

**Table 3:** Top five selected genotypes for high and low source-sink effects for yield related and fiber quality traits.

Treat.	Source-Sink	2018		2019	
		Yield	Quality	Yield	Quality
**E1**	**Low source-sink effect**	Deltapine15	han2490	dezimian531	2011SS
		chaoyangmian1	103030	jimian10	emian17
		zhong416	zhongmiansuo64	guannong1	ejing1
		zhongmiansuo7	shan70	zhongmiansuo50	lu890
		FM1735	ekangmian6	2011SS	xinluzhong41
	**High source-sink effect**	huiyuan717	liaojinmian6	yumian21	han9609
		liaomian17	103026	xinluzao23	liaomian17
		ningmian22	61930	ganmian2	liaomian6
		PB12-1-8	zhongmiansuo41	xinluzao20	ganmian4
		nongda94-7	ji4025	ganmian3	xinluzhong34
**E2**	**Low source-sink effect**	chaoyangmian1	yumian1	2011SS	ganmian2
		xiazao3	ekangmian6	sumian22	SQ152201
		yishuhong	xinluzhong 47	zhongmiansuo50	xinluzhong41
		102909	zhongmiansuo43	dezimian531	xinluzhong40
		FM1735	Delfos97-047	gangmian2	gangmian2
	**High source-sink effect**	PB12-1-10	ji4025	zhongmiansuo43	ganmian11
		N82	guoxinmian9	zhongmiansuo45	liaomian17
		xinluzao4	zhongchuang88	zhongmiansuo30	shizao2
		jinmian5	Stoneville4B	yumian21	Bejing1
		zhongmiansuo14	zhongmiansuo60	shizao1	zhuangjiahan102
**E3**	**Low source-sink effect**	kezi201	xinluzao15	sumian22	liaomian5
		jimian11	zhongmiansuo64	zhongmiansuo50	ganmian2
		xinluzao26	shan79	JEJS	SQ152201
		FM1735	zhong151222	simian3	6426
		zhong416	zhongmiansuo69	zhong662	dezimian531
	**High source-sink effect**	xiazao2	xinluzao10	xinluzao20	gangmian2
		zhongmiansuo14	61930	yumian21	liaomian6
		ningmian22	103026	xinluzao23	ganmian11
		xinluzhong40	daihongdai	ganmian4	liaomian17
		xinluzao60	zhongmiansuo35	611bo	heishanmian1
**E4**	**Low source-sink effect**	yunzaoN95	lumina2153	bo425	annong121
		liaoyangduanjie	xinluzao15	xinluzhong41	xuzhou219
		zhong416	shan70	sumian12	nongda94-7
		FM1735	baimian17	zhongmiansuo50	jinmian23
		xinluzao3	61995	Stoneville2B	ejing1
	**High source-sink effect**	zhongmiansuo74	edaimian	xinluzao20	huihe36
		kemian4	103026	yumian21	han9609
		shizao2	xia13-7	xinluzao23	heishanmian1
		zhongmiansuo14	xinluzao10	ningmian22	emian16
		yumian1	xiangmian13	zhongmiansuo27	nongken5

## Data Availability

Data supporting the reported results will be available and provided at a reasonable request.

## Supplementary Materials

The following are available online at https://www.mdpi.com/article/10.3390/plants10061147/s1, Supplementary Tables S1 to S9 Over-dispersion parameter estimated by Maximum Likelihood using Generalized Linear Model (GLM) for Fiber quality and yield contributing traits in 355 upland cotton accessions under four treatment Effects (E1-E4) across two years 2018 and 2019. Supplementary Table S10. Eigenvalues contributing to different PCs for studied traits related to Source-Sink Effects across years 2018 and 2019. Supplementary Table S10 Loading Matrix Associated with the principal components for studied traits, treatment effects (E1-E4) and years 2018 and 2019.
